# Knockdown of LncRNA MAPT-AS1 inhibites proliferation and migration and sensitizes cancer cells to paclitaxel by regulating MAPT expression in ER-negative breast cancers

**DOI:** 10.1186/s13578-018-0207-5

**Published:** 2018-02-05

**Authors:** Yiyuan Pan, Yiqi Pan, Yue Cheng, Fan Yang, Zhihan Yao, Ouchen Wang

**Affiliations:** 1 Department of Surgical Oncology, The First Affiliated Hospital of Wenzhou Medical University, Wenzhou, 325000 China; 2 Wenzhou Medical University, Wenzhou, 325000 China

**Keywords:** MAPT-AS1, Cell growth, Invasiveness, Paclitaxel resistance, MAPT, Natural antisense transcript, ER-negative breast cancer

## Abstract

**Background:**

MAPT-AS1, a long non-coding RNA, has not been reported in any previous research about its function in cancers. In this study, we investigated the role of MAPT-AS1 in the progression and paclitaxel resistance in breast cancer, and the regulation between MAPT-AS1 and its natural comparable sense transcripts MAPT.

**Methods:**

We analysed the breast cancer patients’ clinical information and explored the function of MAPT-AS1 by gain- and loss-of function assays in vitro and in vivo. The regulation between MAPT-AS1 and MAPT was confirmed by gene expression analysis and rescue assays. To verify the hypothesis that MAPT-AS1 and MAPT might form a duplex structure, we performed RT-PCR assays on RNA after α-amanitin treatment.

**Results:**

By analysing the breast cancer patients’ clinical information from the TCGA database, we found that ER-negative patients with younger age (< 60), larger tumors (≥ 2 cm), metastatic lymph nodes and stages (III–IV) had higher expression of MAPT-AS1. MAPT-AS1 is correlated with the cell growth, invasiveness and paclitaxel resistance by regulating its natural comparable sense transcripts MAPT in ER-negative breast cancer cells. The result revealed that MAPT-AS1 overexpression could partially protect the MAPT mRNA from degradation, while MAPT-AS1 knockdown decreased the stability of MAPT mRNA. Meanwhile, MAPT knockdown decreased the expression of MAPT-AS1 mRNA. MAPT-AS1 expressed coordinately with MAPT in breast tumor tissues.

**Conclusion:**

Our study is the first to report a novel lncRNA MAPT-AS1 in human cancer. ER-negative patients with younger age (< 60), larger tumors (≥ 2 cm), metastatic lymph nodes and stages (III–IV) had higher expression of MAPT-AS1. MAPT-AS1 is correlated with the cell growth, invasiveness and paclitaxel resistance in ER-negative breast cancer cells through antisense pairing with MAPT. MAPT-AS1 may serve as a potential therapeutic target in ER-negative breast cancers.

**Electronic supplementary material:**

The online version of this article (10.1186/s13578-018-0207-5) contains supplementary material, which is available to authorized users.

## Background

Breast cancer is one of the most prevalent cancers in women worldwide with approximately 272,700 newly patients and 61,500 estimated deaths in China in 2012 [1, 2]. In past years, serious efforts have been made to advance the diagnosis, and the disease can be treated by radical surgery and adjuvant therapies. However, breast tumors are diverse in their characteristic molecular features and responsiveness to treatments [3–5]. Long non-coding RNAs (lncRNAs) are defined as non-protein-coding RNAs > 200 nucleotides [6]. At the start, LncRNAs were regarded as the noise of transcription [7]. To date, lncRNAs have gained the widespread attention, as they are reported to drive a variety of cancer phenotypes through their function in the regulation of gene interactions and biological processes [8–10]. A large number of studies have identified the tumorigenic role of lncRNAs in breast cancer. For instance, Zhang et al. found that lncRNA hoax-as2 works as an oncogene and has a significant role as a potential prognostic and therapeutic target in breast cancer [11, 12]. Xu et al. also identified that lncRNAs can promote cell proliferation and metastasis; therefore, they can serve as a biomarker to diagnose and treat breast cancer in China [13]. In our unpublished studies, we performed the whole-transcriptome sequencing of 23 pairs of breast cancer tumor samples and adjacent non-tumorous tissues, and MAPT-AS1 was originally identified. However, the function of lncRNA MAPT-AS1 remained unclear in cancers. In this study, we performed a series of in vivo and in vitro studies to explore the role and mechanism of lncRNA MAPT-AS1 in breast cancer.

## Methods

### Cell culture

All the breast cell lines were purchased from the Chinese Academy of Sciences (Shanghai, China). The MDA-MB-231 cells were maintained in DMEM culture medium with 10% FBS (Gibco) at 37 °C with 5% CO_2_. The BT-549 and SK-BR-3 cells were maintained in 1640 culture medium with 10% FBS (Gibco) at 37 °C with 5% CO_2_. The MDA-MB-468 and MDA-MB-436 cells were cultured in L-15 (Invitrogen, USA) with 10% FBS (Gibco) at 37 °C with no CO_2_. MCF-10A cells were cultured in DMEM/F12 media with 5% horse serum, 0.5 μg/ml hydrocortisone, 10 μg/ml insulin, 20 ng/ml EGF, 50 units/ml penicillin, 50 μg/ml streptomycin, 100 ng/ml cholera toxin, and 2 mM l-glutamine at 37 °C with 5% CO_2_.

### RNA extraction and quantitative real-time polymerase chain reaction (qRT-PCR)

The total RNA of the cultured cells was isolated by TRIzol reagent (Life Technologies, Carlsbad, CA) according to the manufacturer’s instructions. The cytoplasmic and nuclear RNA were isolated and purified by Cytoplasmic and Nuclear RNA Purification Kit (Norgen, Belmont, CA, USA). The RNA was reversely transcribed into cDNA using the PrimeScript RT Master Mix (Takara, Cat. #RR036A). Real-time PCR was performed with SYBR Premix Ex Taq II (Takara, Cat. #RR820A). The result was normalized to the expression of GAPDH. The primers sequences are as follows: MAPT-AS1, forward GGAGCTTGGCAGTCCAGGTT and reverse CAGAGACACACAGGGAGAATGC; MAPT: forward TCTCACACTGGCTCCAGACACA and reverse CCCGACCTCGTGGCTTTACTTG.

### Transient transfection

The small interference RNA (siRNA) targeting MAPT-AS1, MAPT and plasmid vectors encoding the full MAPT-AS1 sequences was synthesized by GenePharma Co. (Shanghai China). The sequences of si-RNA are as follows: MAPT-AS1-1(siRNA-1): forward 5′-CCCAUGAUGGAGUAGAUUUTT-3′ and reverse 5′-AAAUCUACUCCAUCAUGGGTT-3′. MAPT-AS1-2(siRNA-2), forward 5′-GCACUAUGGACUGUUACAATT-3′ and reverse 5′-UUGUAACAGUCCAUAGUGCTT-3′. MAPT, forward 5′-GCCAGGAGUUCGAAGUGAUTT-3′ and reverse 5′-AUCACUUCGAACUCCUGGCTT-3′. The tumor cells were transfected with siRNA using RNAiMAX or transfected with a plasmid vector using Lipofectamine 3000. The cells were harvested for the subsequent analysis 48 h after transfection.

### Cell proliferation and drug sensitivity assay

The CCK-8 assay was used to detect the cell proliferation and drug sensitivity. For the cell proliferation, the treated tumor cells (5 × 10^3^ cells/well) were seeded into 96-well plates with 5 replicates and incubated for 24, 48, 72 h at 37 °C with 5% CO_2_ for MDA-MB-231 or no CO_2_ for MDA-MB-468. For colony formation, the transfected tumor cells (5 × 10^3^ cells/well) were seeded into 6-well plates with 3 replicates and incubated in media containing 10% FBS for 10 days. The cells were fixed with 4% paraformaldehyde for 30 min and stained with crystal violet for 30 min.

For the drug sensitivity assay, the transfected tumor cells (5 × 10^3^ cells/well) were planted into 96-well plates with 5 replicates for 24 h, then different concentrations of paclitaxel were added into the indicated wells and the cells were cultured for 24 h. Then 10 µl of CCK-8 reagent was added to each well. The cells were incubated for 3 h before recording the absorbance at 450 nm.

### Transwell assay

For migration, the Transwell cell culture chambers (Corning Costar Corp, Cambridge, MA, USA) were used. The two transfected cells were seeded into the upper chamber with serum-free medium, while the bottom chamber was filled with 600 ml 10% FBS medium. For invasion, the BioCoat™ Matrigel Invasion Chamber 24-Well Plate 8.0 Micron (Corning, NY, USA) was used. The two transfected cells were seeded into the upper chamber with serum-free medium, while the bottom chamber was filled with 600 ml 20% FBS medium. The MDA-MB-231 plates were incubated at 37 °C with 5% CO_2_, and the MDA-MB-468 plates were incubated at 37 °C with no CO_2_. After 24 h, the indicated cells were fixed with 4% PFA (Sigma) for 30 min, washed with PBS, then stained with 0.01% crystal violet for 20 min, and then finally air dried.

### Protein extraction and western blot

The breast cells were transfected with siRNAs, and the vectors were cultured for 72 h. Then each whole cell protein was extracted from the cell lines using RIPA lysis buffer. MAPT were used as the detected antibodies. GAPDH was selected as the housekeeping gene. The protein was detected using a SuperSignal protein detection kit (Pierce, Rockford, IL).

### Apoptosis detection

The treated MDA-MB-468 and MDA-MB-231 cells were digested with trypsin without EDTA, washed with PBS three times, and then stained with Annexin V-FITC and propidium iodide (PI). Apoptotic cells were measured using a flow cytometer (FACScan, BD Biosciences) equipped with CellQuest software (BD Biosciences) following the manufacturer’s instructions.

### In vivo nude mouse models

Female 5-week old nude mice were raised in the experimental animal center of Wenzhou Medical University. The MDA-MB-231 cells (100 × 10^4^) were injected into the subcutaneous fat of the mice and maintained for 14 days. The tumors were confirmed to grow successfully. Then the mice were divided randomly into 2 groups and injected with siRNA-Invivofectamine 2.0 complexes (100 μl of 0.5 mg/ml relative to siRNA) directly into xenograft tumors as previously described [14] at 14, 17, 20 days. The tumor volume was measured at 8, 11, 14, 17, 20 and 23 days using the equation vol = (length (cm)  ×  width (cm)  ×  width (cm)  ×  0.5326). The mice were sacrificed at day 23 and the tumors were removed, weighed and photographed.

### Statistical analysis

The results of three independent experiments were presented as the mean ± standard deviations (SD). An unpaired Student’s t test was used to calculate the difference between the treatment groups and control groups with GraphPad software version 5.0 (GraphPad Software, CA, USA). P < 0.05 was considered statistically significant.

## Results and discussion

### MAPT-AS1 is a bona fide lncRNA and a natural antisense of MAPT

**Fig. 1:**
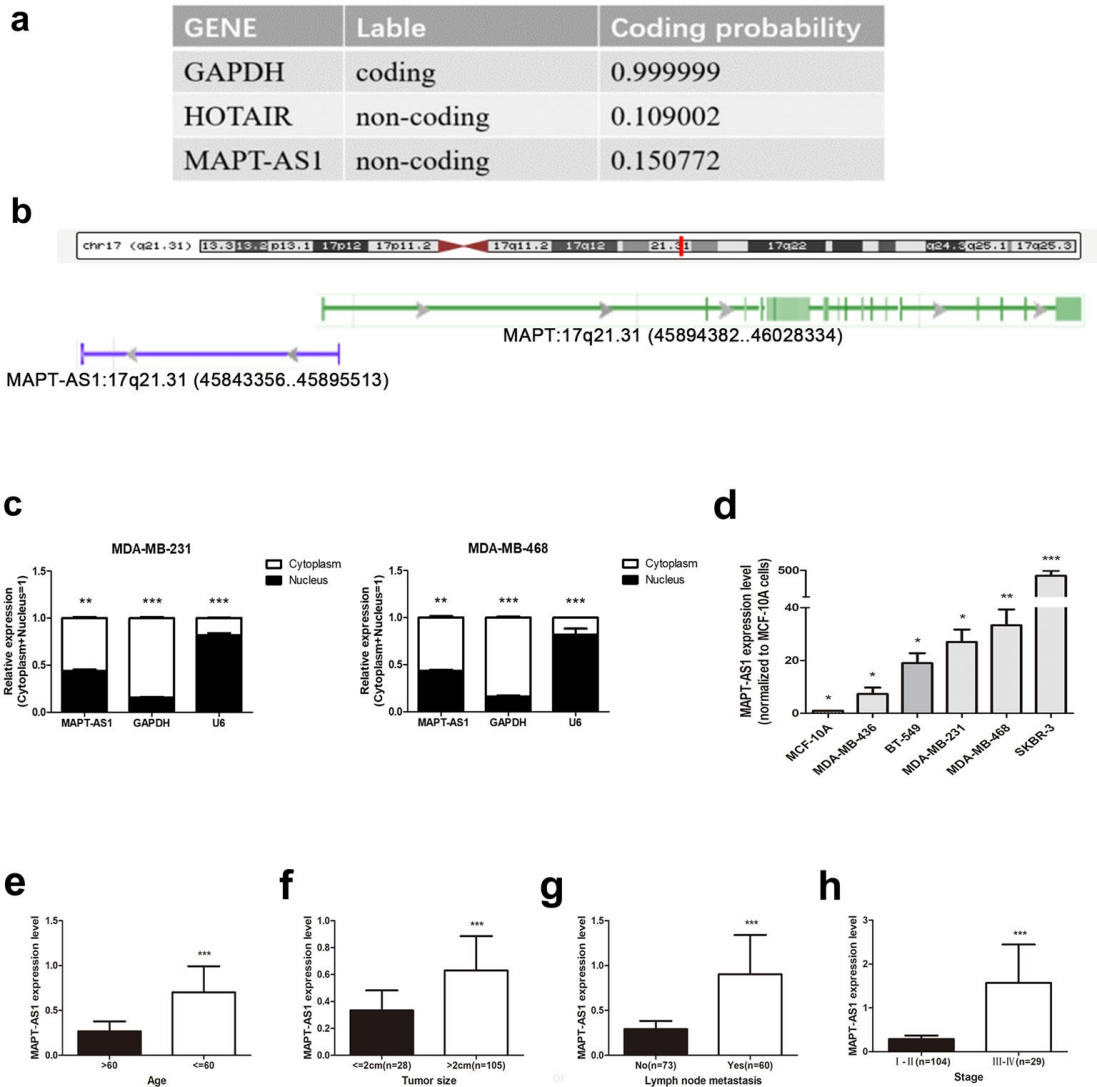
**a** Coding potential calculator 2 (CPC 2.0) was used to predict GAPDH, HOTAIR and MAPT-AS1′ protein-coding probability. **b** MAPT-AS1 is an 840 bp RNA transcribed from chromosome 17, the opposite strand of MAPT (17q21.31). **c** MAPT-AS1 levels in the nucleus and cytoplasmic compartments of MDA-MB-231 and MDA-MB-468 cells were detected using qRT-PCR. GAPDH was used as the cytoplasmic control, and U6 snoRNA was used as the nuclear control. **d** MAPT-AS1 expression level was determined in the ER-negative breast cell lines with qRT-PCR. **e**–**h** ER-negative patients with younger age (< 60), larger tumors (≥ 2 cm), metastatic lymph nodes and stages (III–IV) had higher expression of MAPT-AS1

First of all, to prove that MAPT-AS1 is a lncRNA, Coding Potential Calculator 2 (CPC 2.0) was used to predict its protein-coding probability. CPC 2.0 owns an overall accuracy of 0.961, specificity of 0.970 and sensitivity of 0.952 [15]. The result showed that MAPT-AS1 had a low coding probability (Fig. 1a). MAPT-AS1 is an 840 bp RNA transcribed from chromosome 17, the opposite strand of MAPT (17q21.31) [16], and it consists of two exons (Fig. 1b). To detect the subcellular localization of MAPT-AS1, we fractionated MDA-MB-231 and MDA-MB-468 cells into nuclear and cytoplasmic fractions. The result showed that MAPT-AS1 molecules were located more within the cytoplasm than the nucleus (Fig. 1c).

### MAPT-AS1 was related to the progression in ER-negative breast cancers

In our unpublished studies, we performed the whole-transcriptome sequencing of 23 pairs of breast cancer tumor samples and adjacent non-tumorous tissues, and MAPT-AS1 was originally identified. We detected the expression levels of 5 breast cancer cells, and the normal epithelial cell line MCF-10A was used as the control. The data confirmed that MAPT-AS1 was overexpressed in breast cancer cells compared to MCF-10A (Fig. 1d). By analysing the breast cancer patients’ clinical information from the TCGA database, we found that ER-negative patients with younger age (< 60), larger tumors (≥ 2 cm), metastatic lymph nodes and stages (III–IV) had higher expression of MAPT-AS1 (Fig. 1e–h). However, there was no such correlation in ER-positive patients. These results revealed that MAPT-AS1 might be related to the progression in ER-negative breast cancers. We also found that MAPT-AS1 expressed higher in ER-positive tumor tissues and cells than ER-negative tumor tissues and cells (Additional file 1: Fig. S1a, b).

### MAPT-AS1 was correlated with migration, invasion, and proliferation

**Fig. 2:**
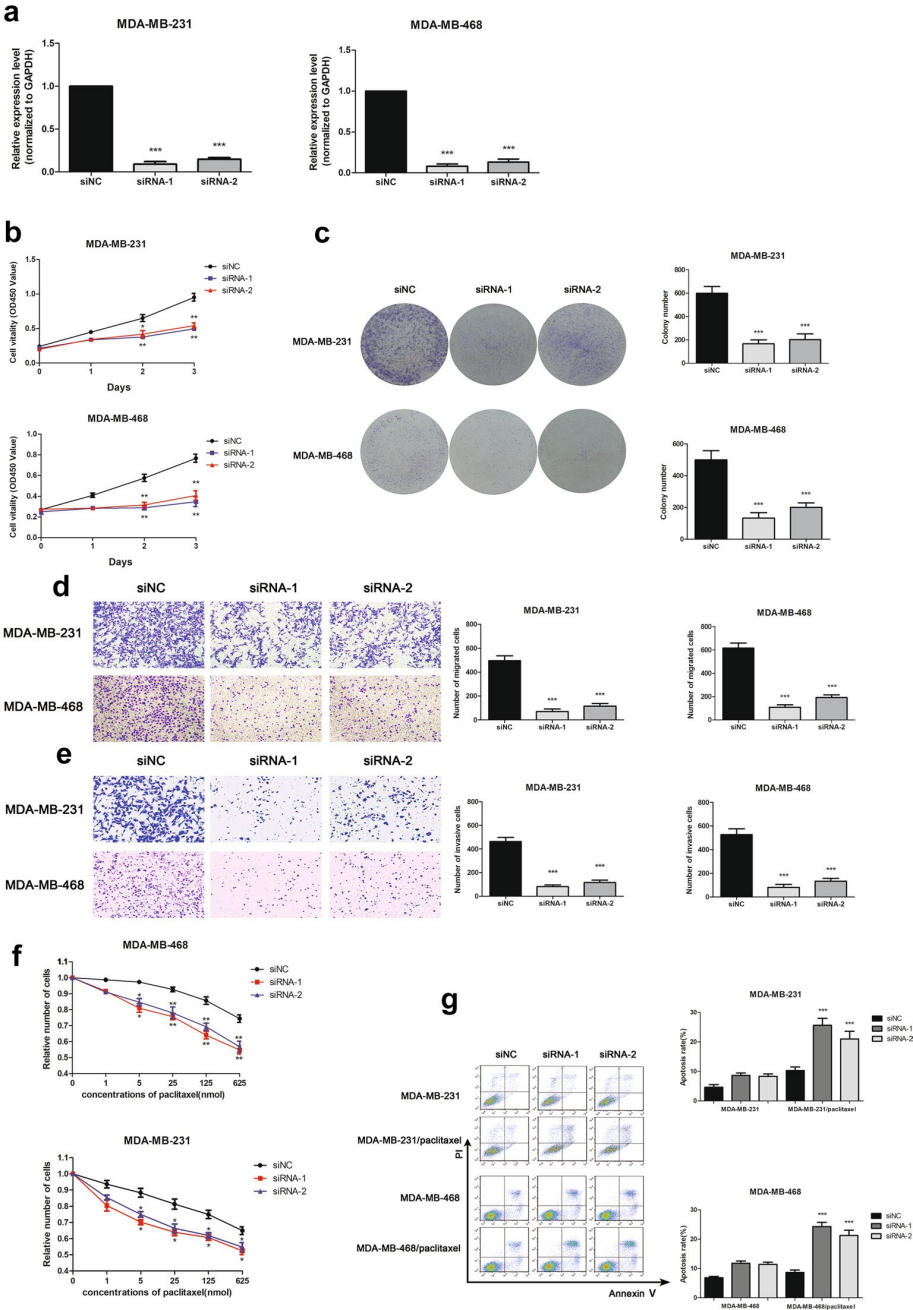
**a** The MAPT-AS1 expression level was determined by qRT-PCR in MDA-MB-231 and MDA-MB-468 cells transfected with siRNAs. GAPDH was used as control. **b**, **c** The MDA-MB-231 and MDA-MB-468 cells were transfected with siRNA-1/2 or negative control for 48 h. The relative cell proliferation was measured using CCK-8 assays and colony-forming assays. **d**, **e** The migration and invasion ability of the two cell lines transfected with siRNA-1/2 or NC were measured by Transwell assays. **f**, **g** The two cell lines transfected with siRNA-1/2 or NC were then treated with paclitaxel for 24 h before CCK-8 assays or apoptosis detection. The apoptosis rate were measured using flow cytometry assays

**Fig. 3:**
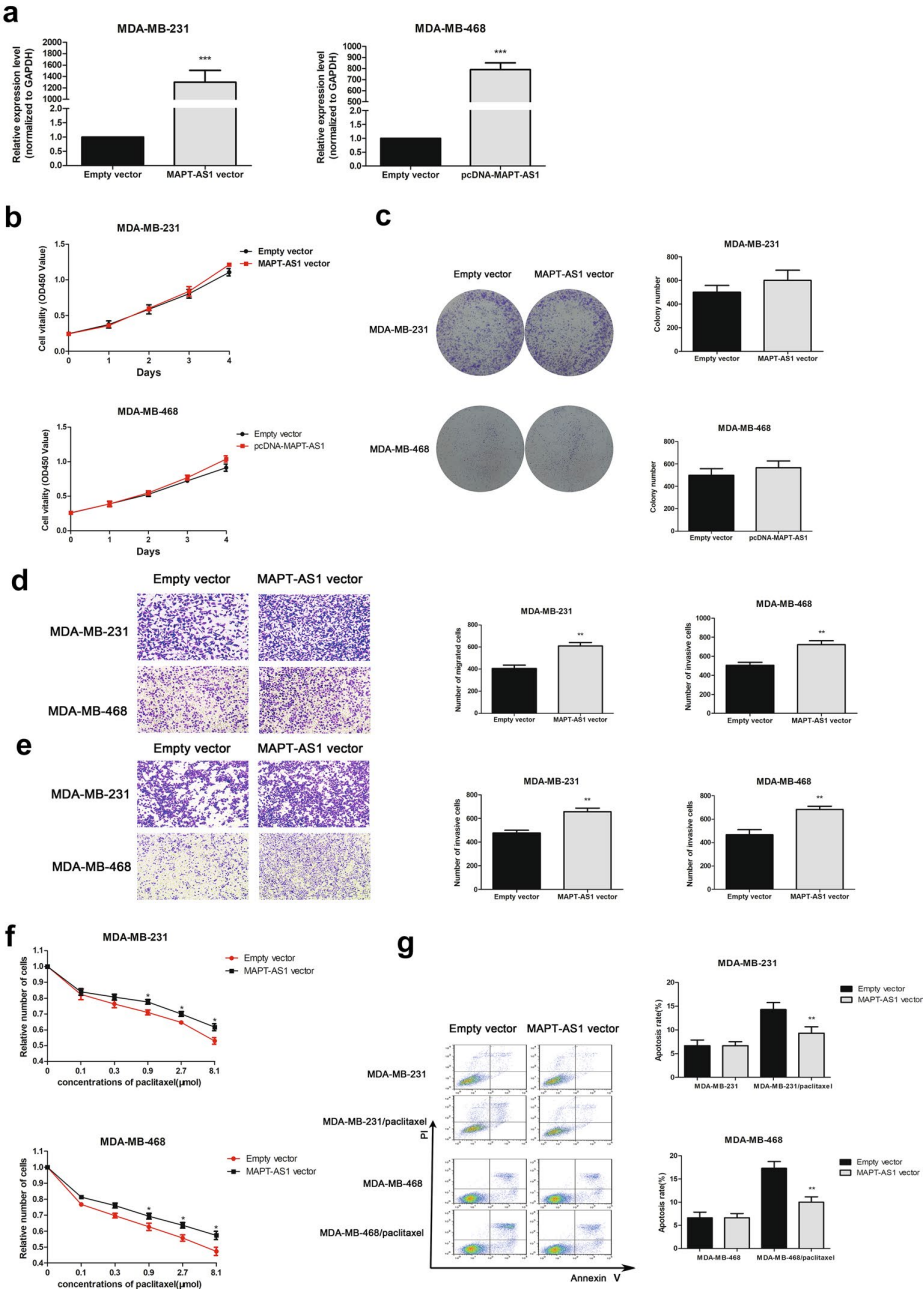
**a** The MAPT-AS1 expression level was determined by qRT-PCR in MDA-MB-231 and MDA-MB-468 cells transfected with MAPT-AS1 vector or empty vector. GAPDH was used as control. **b**, **c** The MDA-MB-231 and MDA-MB-468 cells were transfected with MAPT-AS1 vector or empty vector for 48 h. The relative cell proliferation was measured using CCK-8 assays and colony-forming assays. **d**, **e** Transwell assays were used to measure the migration and invasion ability of the two cell lines transfected with MAPT-AS1 vector or empty vector. **f**, **g** The two cell lines transfected with MAPT-AS1 vector or empty vector were then treated with paclitaxel for 24 h before CCK-8 assays or apoptosis detection. The apoptosis rate were measured using flow cytometry assays

We knocked down the MAPT-AS1 expression with targeted siRNAs in MDA-MB-231 and MDA-MB-468 cells, and overexpressed MAPT-AS1 using plasmid vectors encoding the full MAPT-AS1 sequences in MDA-MB-231 and MDA-MB-468 cells. The qRT-PCR assays showed that MAPT-AS1 was effectively downregulated by the targeting siRNAs (Fig. 2a, p < 0.01), whereas MAPT-AS1 was highly upregulated using plasmid vectors compared with the control cells (Fig. 3a, p < 0.01).

To test the growth effect of MAPT-AS1, we performed CCK-8 and colony formation assays on the cells. The date revealed that the proliferation rates and colony numbers significantly decreased in the MAPT-AS1-knockdown cells (Fig. 2b, c), and increased in the MAPT-AS1-overexpression cells compared to the control groups (Fig. 3b, c).

We performed migration and invasion assays to examine the effect of MAPT-AS1 in the cell deformability. The result showed that knockdown of MAPT-AS1 dramatically inhibited the migration and invasion of both MDA-MB-231 and MDA-MB-468 cells (Fig. 2d, e), while overexpression of MAPT-AS1 led to promoting migration and invasion in the cells (Fig. 3d, e) compared with the control groups.

### MAPT-AS1 is correlated with the paclitaxel sensitivity

As the research revealed, lncRNA could affect the paclitaxel resistance of cancer cells [17, 18]; we suspected that MAPT-AS1 might be involved in the sensitivity to paclitaxel in breast cancer cells. To confirm the hypothesis, we set a series of increasing concentrations of paclitaxel. As shown in Fig. 2f, the MAPT-AS1-knockdown cells were significantly more sensitive to paclitaxel; conversely, overexpression of MAPT-AS1 led to decreased paclitaxel sensitivity of the cells compared to the control groups (Fig. 3f). Flow cytometry analysis showed that the apoptotic rates were markedly upregulated in MAPT-AS1 knockdown cells (Fig. 2g) while downregulated in MAPT-AS1 overexpressed cells after treatment with paclitaxel (Fig. 3g).

### MAPT-AS1 silencing inhibited the tumorigenesis of breast cancer cells in nude mice

**Fig. 4:**
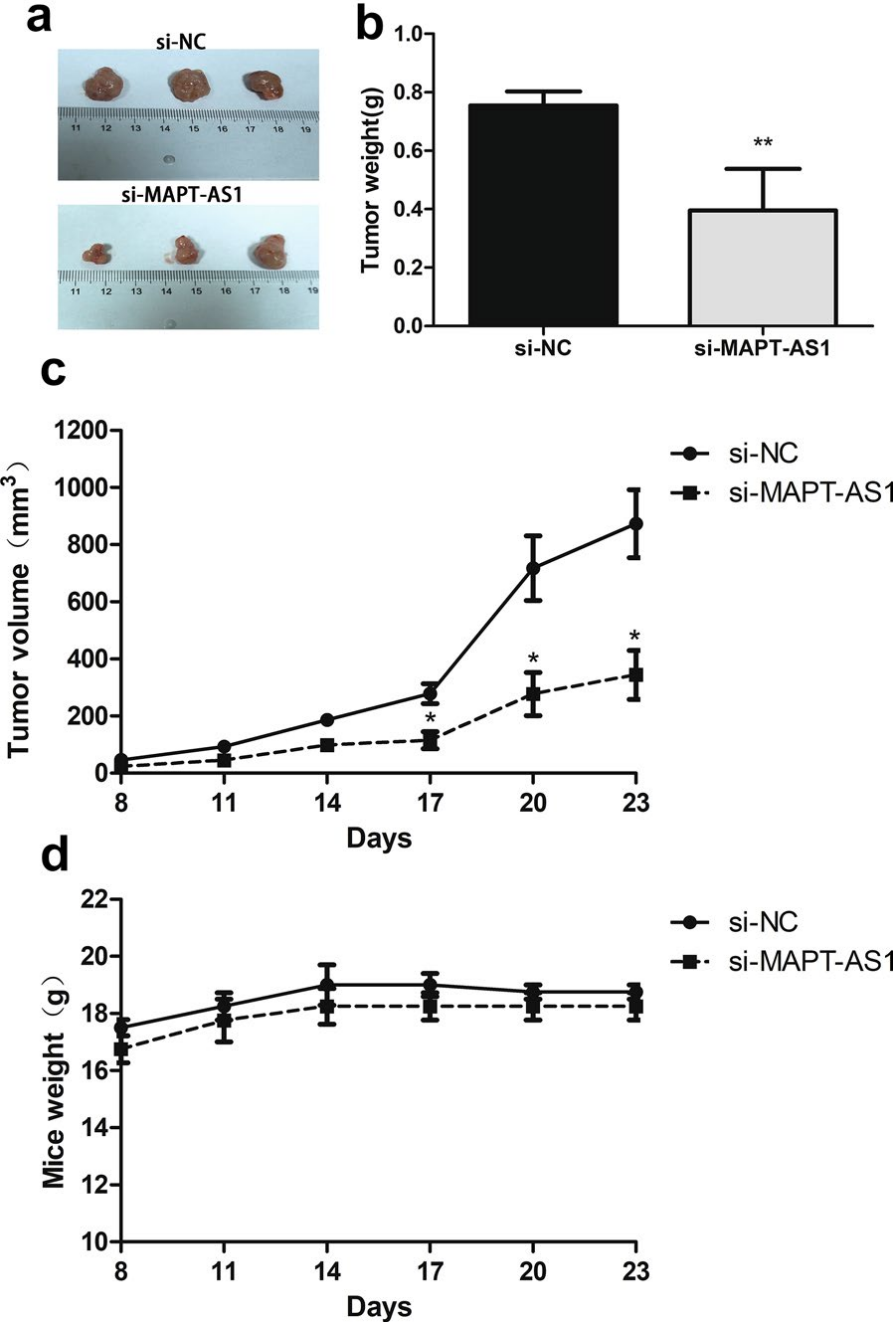
**a** Tumors dissected from the sacrificed nude mice. **b**, **c** The mice were injected with siRNA-Invivofectamine 2.0 complexes (100 μl of 0.5 mg/ml relative to siRNA) directly into xenograft tumors as previously described at 14, 17, 20 days. The tumor weight and volume were measured and compared at 8, 11, 14, 17, 20 and 23 days. **d** Body weights were monitored daily

All of the nude mice with subcutaneously injected stably transfected MDA-MB-231 cells developed xenograft tumors, and the tumor sizes of the nude mice were measured every week. The results showed that MAPT-AS1 knockdown markedly suppressed the tumor growth rates (Fig. 4a–c), while the mice weight was not affected (Fig. 4d).

### MAPT-AS1 was correlated with MAPT expression

**Fig. 5:**
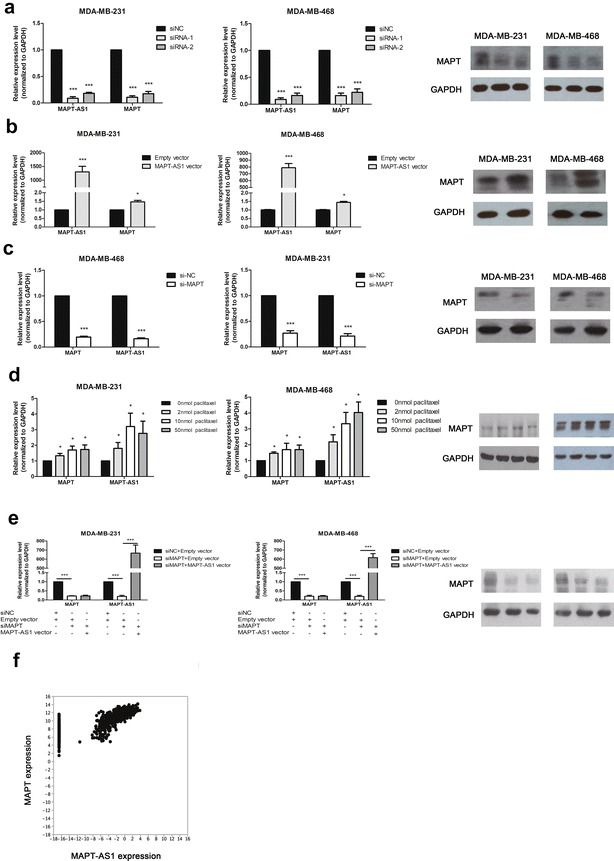
**a** The MAPT expression level was determined by qRT-PCR and western blot assays in MDA-MB-231 and MDA-MB-468 cells transfected with siRNA-1/2 or negative control. **b** The MAPT expression level was determined by qRT-PCR and western blot assays in the two cell lines transfected with MAPT-AS1 vector or empty vector. **c** The MAPT-AS1 expression level was measured by qRT-PCR in MDA-MB-231 and MDA-MB-468 cells transfected with si-MAPT or negative control. **d** The MAPT expression level was detected by qRT-PCR and western blot assays in the two cell lines after treated with different concentrations of paclitaxel for 24 h, while the MAPT-AS1 expression level was measured by qRT-PCR. **e** The MDA-MB-231 and MDA-MB-468 cells were pretreated with si-MAPT, then transfected with MAPT-AS1 vector or empty vector. The MAPT expression level was determined by qRT-PCR and western blot assays. **f** The MAPT and MAPT-AS1 expression levels in breast tumor tissues from TCGA database

We performed PCR and Western Blot to confirm the relationship between MAPT-AS1 and MAPT, and the result showed that knockdown of MAPT-AS1 significantly decreased the expression of MAPT both in mRNA and the protein levels (Fig. 5a) while overexpression of MAPT-AS1 could upregulate the expression of MAPT (Fig. 5b). Meanwhile, knockdown of MAPT also decreased the MAPT-AS1 expression in both cells (Fig. 5c). However, overexpression of MAPT-AS1 could not increase the expression of MAPT in the cells pretreated with si-MAPT both in mRNA and the protein levels (Fig.5e). Furthermore, it was confirmed by the TCGA database that MAPT-AS1 was expressed coordinately with MAPT in breast tumor tissues (Fig. 5f).

### Paclitaxel induced the expression of MAPT-AS1 and MAPT

To determine whether upregulation of MAPT-AS1 and MAPT were functionally needed in paclitaxel tolerance, we cultured the tumor cells in low concentrations of paclitaxel for 24 h, and the results showed that the expression of MAPT-AS1 and MAPT were moderately elevated (Fig. 5d).

### MAPT overexpression rescued the si-MAPT-AS1-induced tumorigenesis inhibition

**Fig. 6:**
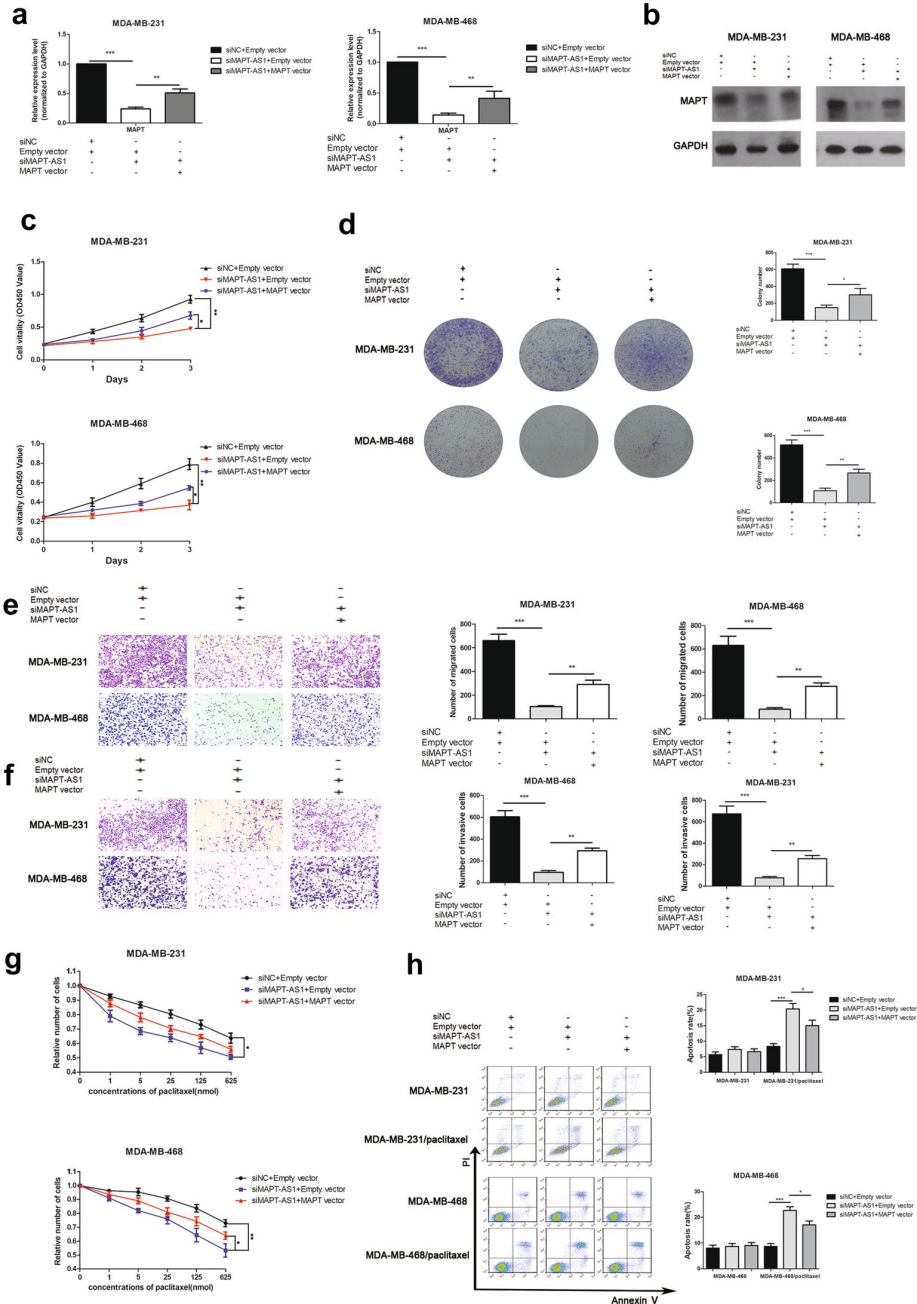
**a**, **b** The MDA-MB-231 and MDA-MB-468 cells were pretreated with si-MAPT-AS1, then transfected with MAPT vector or empty vector. The MAPT expression level was determined by qRT-PCR and western blot assays. GAPDH was used as control. **c**–**f** The two cell lines pretreated with si-MAPT-AS1 were then transfected with MAPT vector or empty vector. The relative cell proliferation was measured using CCK-8 assays and colony-forming assays. And the migration and invasion ability were measured by Transwell assays. **g**, **h** The two cell lines pretreated with si-MAPT-AS1 were then transfected with MAPT vector or empty vector, and further exposed to paclitaxel for 24 h before CCK-8 assays or apoptosis detection. The apoptosis rate were measured using flow cytometry assays

Our previous results demonstrated that MAPT-AS1 was regulated and expressed coordinately with MAPT, so it was possible that the MAPT overexpression might rescue the si-MAPT-AS1-induced tumorigenesis inhibition. As shown in Fig. 6, MAPT-AS1 knockdown led to marked tumorigenesis inhibition and increased the paclitaxel sensitivity in breast cancer cells, while simultaneous MAPT overexpression was able to reverse the tumorigenesis inhibition and upregulated paclitaxel resistance.

### MAPT and MAPT-AS1 might form an RNA–RNA duplex structure

**Fig. 7:**
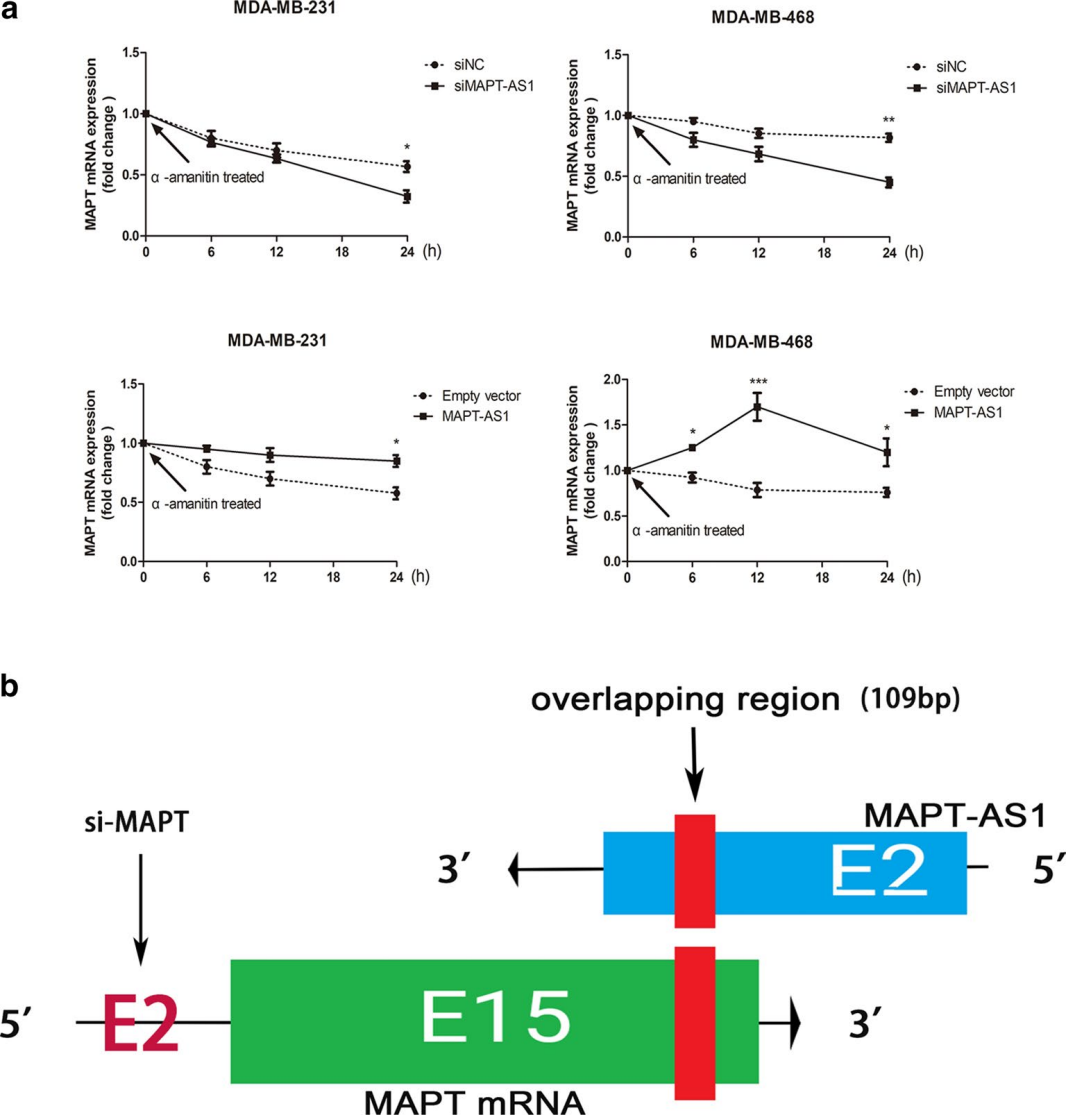
**a** Stability of MAPT mRNA over time was measured by qPCR after treatment with α-amanitin (100 nM). The MDA-MB-231 and MDA-MB-468 cells were transfected with si-MAPT-AS1, si-NC, MAPT-AS1 vector or empty vector. The transfected cell lines were further exposed to 100 nM α-amanitin for 6, 12 or 24 h. 18S RNA was used as a control. **b** Freiburg RNA Tools were used to predict the overlapping region

RNA duplex formation might change the secondary or tertiary structure of MAPT mRNA, and protect it from RNase degradation [19, 20]. To verify the hypothesis that MAPT-AS1 and MAPT might form a duplex structure, we performed RT-PCR assays on RNA from MDA-MB-468 and MDA-MB-231 cells. For the control, 18s ribosomal RNA, which was not affected by α-amanitin treatment, was used. The result revealed that MAPT-AS1 overexpression could partially protect the MAPT mRNA from degradation, while MAPT-AS1 knockdown decreased the stability of MAPT mRNA (Fig. 7a). From the above, our data demonstrates that MAPT-AS1 upregulated the stability of MAPT mRNA in breast cancer cells.

However, overexpression of MAPT-AS1 could not increase the expression of MAPT in the cells pretreated with si-MAPT, which aimed at the exon 2 of MAPT mRNA. We used Freiburg RNA Tools to predict the overlapping region, and the results showed that MAPT-AS1 and MAPT might form an duplex structure at the exon15 of MAPT mRNA (Fig. 7b).

## Discussion

As the main cause of cancer-related deaths in women, breast cancer holds a low 5-year survival rate for patients with metastasis or those resistant to paclitaxel, which is the first-line chemotherapy drug for breast cancer. Thus, there is an urgent need to find the underlying mechanism and a therapeutic target for these patients. In our unpublished studies, we found a novel lncRNA named MAPT-AS1, which was further confirmed to be highly upregulated in breast tumor tissues compared to the adjacent normal tissues in the TCGA database. However, the detailed mechanism of MAPT-AS1 in the progression of the cancers has not been investigated yet. In this study, we determined that MAPT-AS1 was significantly correlated with breast cancer cells’ biological behaviors in vitro and in vivo. Knockdown of MAPT-AS1 markedly inhibited tumorigenicity and upregulated the sensitivity to paclitaxel, thus indicating it may be a novel therapeutic target in breast cancers.

MAPT-AS1 is the natural antisense transcript of MAPT. Natural antisense transcripts are common in cells and most transcribed regions have antisense transcripts [21]. However, the mechanism of how the majority of antisense lncRNAs regulate the comparable sense transcripts still remains unknown. Here, we confirmed that MAPT-AS1 is concordantly upregulated with its sense transcript, MAPT, in tumor tissues. Knockdown of MAPT-AS1 markedly decreased the MAPT mRNA and protein level, while overexpression of MAPT-AS1 increased the MAPT mRNA and protein level.

MAPT, encoded by a single copy gene on chromosome 17q2121 [22], expresses eight isoforms by alternative mRNA splicing, and its protein is called TAU. MAPT expression is upregulated by estrogen in vitro and in vivo [23, 24], and our results showed MAPT-AS1 expressed coordinately with MAPT, thus it was common that MAPT-AS1 expressed much higher in ER-positive tumor tissues and cells.

In these years, MAPT was reported to be associated with metastasis and paclitaxel resistance in various cancers including breast cancer [25–29]. TAU can promote the assembly of tubulin dimers into microtubules to maintain the stability of microtubule complexes [30], and TAU promotes microtentacle formation and increased the reattachment of suspended cells [29]. Our results showed that high MAPT-AS1 expression were clinically inclined to have larger tumors, metastatic lymph nodes and stages (III–IV) in the ER-negative tumors. The previous studies also showed that TAU competes against paclitaxel to bind the same domain at the microtubules [31]. Thus, low TAU expression can render microtubules more vulnerable to paclitaxel-induced damage [32].

Knockdown of MAPT-AS1 markedly decreased the expression of TAU, thereby inhibiting the assembly of tubulin dimers into microtubules and microtentacle formation, and weakening its ability to compete against paclitaxel, finally inhibited the proliferation and migration and sensitizes the breast cancer cells to paclitaxel. Meanwhile, overexpression of MAPT-AS1 markedly upregulated the expression of TAU, thus increasing the microtentacle formation and enhancing its ability to compete against paclitaxel, finally promoted the migration and decreased sensitivity to paclitaxel in the breast cancer cells. As for the reason why upregulated expression of TAU didn’t promote the proliferation of cancer cells, we hypothesized that the amount of TAU in breast cancer cells may be enough for the assembly of tubulin dimers into microtubules to maintain the stability of microtubule complexes. When we upregulated expression of TAU, the surplus of TAU didn’t make a big difference in the assembly of tubulin dimers into microtubules. Further research needs to be performed to confirm our hypothesis.

We further showed that MAPT-AS1 and MAPT may form an RNA–RNA duplex. Although it is difficult to confirm the endogenous RNA–RNA duplexes, we used α-amanitin to inhibit the eukaryotic RNA polymerase, especially polymeraseIItranscription. The results showed that the stability of MAPT mRNA was upregulated after α-amanitin treatment. MAPT-AS1 and MAPT might form an RNA–RNA duplex at their overlapping region and upregulate the stability of MAPT mRNA by changing the secondary or tertiary structure of MAPT mRNA. It has been reported that antisense transcripts could compete with microRNAs, which bind the same target genes, thus increasing the mRNA expression level [33, 34]. There are eight isoforms in MAPT mRNAs. We analysed the isoforms and found that all isoforms contain exon15, which is 4380 bp, much more longer than any other exon. We used Freiburg RNA Tools to predict the overlapping region, while the underlying mechanism still requires further exploration.

In conclusion, our study is the first to report a novel lncRNA MAPT-AS1 in human cancer, and provide solid evidence of the regulation between MAPT-AS1 and MAPT in breast cancer cells. By analysing the ER-negative patients’ clinical information, we found that patients with younger age (< 60), larger tumors (≥ 2 cm), metastatic lymph nodes and stages (III–IV) had higher expression of MAPT-AS1. Our study confirmed that knockdown of LncRNA MAPT-AS1 inhibites proliferation and migration and sensitizes cancer cells to paclitaxel through antisense pairing with MAPT. MAPT-AS1 may serve as a potential therapeutic target in ER-negative breast cancers.

## Additional file

**Additional file 1: Figure S1.** a MAPT-AS1 expression in breast cancer patients from the TCGA database. b MAPT-AS1 expression level was determined in the breast cell lines by qRT-PCR.
