# Supplementary figures and images for: Knockdown of LncRNA MAPT-AS1 inhibites proliferation and migration and sensitizes cancer cells to paclitaxel by regulating MAPT expression in ER-negative breast cancers
Source: Cell Biosci. 2018 Feb 5;8:7. doi: 10.1186/s13578-018-0207-5 (PMC5799917; doi:10.1186/s13578-018-0207-5)

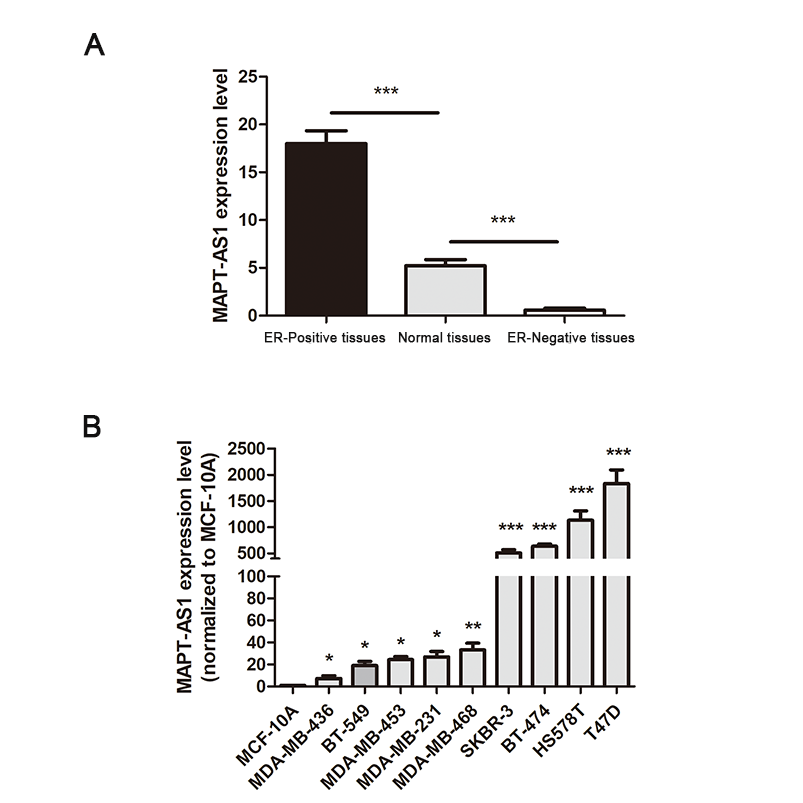

Supplement: Supplementary file 1 — Additional file 1: Figure S1. a MAPT-AS1 expression in breast cancer patients from the TCGA database. b MAPT-AS1 expression level was determined in the breast cell lines by qRT-PCR. [file 13578_2018_207_MOESM1_ESM.tif]
